# Respiratory tract infection-related healthcare utilisation in children with Down’s syndrome

**DOI:** 10.1007/s15010-020-01408-5

**Published:** 2020-03-14

**Authors:** Logan Manikam, Anne G. M. Schilder, Monica Lakhanpaul, Peter Littlejohns, Emma C. Alexander, Andrew Hayward

**Affiliations:** 1 UCL Institute of Epidemiology and Healthcare, University College London, 1-19 Torrington Place, London, WC1E 6BT UK; 2 UCL Institute of Health Informatics Research, University College London, 222 Euston Road, London, NW1 2DA UK; 3 National Institute of Health Research University College London Hospitals Biomedical Research Centre, 149 Tottenham Court Road, London, W1T 7DN UK; 4 evidENT, UCL Ear Institute, University College London, 332 Grays Inn Road, London, WC1X 8DA UK; 5 Population, Policy & Practice, UCL Great Ormond Street Institute of Child Health, University College London, 30 Guilford Street, London, WC1N 1EH UK; 6 Whittington Health NHS Trust, Magdala Avenue, London, N19 5NF UK; 7 Centre for Implementation Science, Institute of Psychiatry, Psychology and Neurosciences, King’s College London, 6 De Crespigny Park, Camberwell, London, SE5 8AB UK; 8 Paediatric Liver, GI and Nutrition Centre and Mowatlabs, King’s College Hospital, Denmark Hill, London, SE5 9RS UK

**Keywords:** Respiratory, Down’s syndrome, Infections, Antibiotics, Hospitalisation, Community

## Abstract

**Purpose:**

Children with Down’s syndrome (DS) are prone to respiratory tract infections (RTIs) due to anatomical variation, immune system immaturity and comorbidities. However, evidence on RTI-related healthcare utilisation, especially in primary care, is incomplete. In this retrospective cohort study, we use routinely collected primary and secondary care data to quantify RTI-related healthcare utilisation in children with DS and matched controls without DS.

**Methods:**

Retrospective cohort study of 992 children with DS and 4874 matched controls attending English general practices and hospitals as identified in Clinical disease research using LInked Bespoke studies and Electronic health Records (CALIBER) from 1997 to 2010. Poisson regression was used to calculate consultation, hospitalisation and prescription rates, and rate ratios. Wald test was used to compare risk of admission following consultation. The Wilcoxon rank–sum test was used to compare length of stay by RTI type and time-to-hospitalisation.

**Results:**

RTI-related healthcare utilisation is significantly higher in children with DS than in controls in terms of GP consultations (adjusted RR 1.73; 95% CI 1.62–1.84), hospitalisations (adjusted RR 5.70; 95% CI 4.82–6.73), and antibiotic prescribing (adjusted RR 2.34; 95% CI 2.19–2.49). Two percent of children with DS presenting for an RTI-related GP consultation were subsequently admitted for an RTI-related hospitalisation, compared to 0.7% in controls.

**Conclusions:**

Children with DS have higher rates of GP consultations, hospitalisations and antibiotic prescribing compared to controls. This poses a significant burden on families. Further research is recommended to characterise healthcare behaviours and clinical decision-making, to optimise care for this at risk group.

**Electronic supplementary material:**

The online version of this article (10.1007/s15010-020-01408-5) contains supplementary material, which is available to authorized users.

## Introduction

Approximately 1 in 1000 children born in England and Wales will be born with Down’s syndrome (DS), or trisomy 21. This equates to around 750 children born with DS every year [1]. DS is, therefore, one of the most common genetic conditions in the United Kingdom. DS has a known association with congenital heart problems, gastrointestinal abnormalities, thyroid dysfunction, and also an increased tendency for respiratory infections (RTIs) [2].

RTIs can be split into upper respiratory tract infections (URTIs), and lower respiratory tract infections (LRTIs). Most URTIs are viral in origin and are commonly due to *rhinoviruses*, *coronaviruses*, *parainfluenza* viruses, respiratory syncytial virus (RSV), *adenoviruses* and *influenza* viruses. In contrast, the aetiology of LRTIs is more mixed with a study of hospitalised children noting a bacterial (25%), viral (25%) or mixed (20%) cause for RTIs [3]. The overall risk of complications is generally low following acute respiratory tract infections [4]. However, the risk of complications is thought to be increased in children with certain comorbidities such as DS.

Important evidence on the burden of respiratory tract infections in children with DS was gathered in Australia. A cohort of 405 children with DS born between 1983 and 1999 was followed up from birth until 2004 [5]. They were hospitalised 3786 times in this period, of which almost one third of admissions was for respiratory tract infections, affecting 52.6% of all children with DS, with an admission rate of 11.4 per 1,000 person years at risk, a rate 17.9 times higher than in the general paediatric population. Similarly, in a USA study of 217 children with DS born between 1997 and 1999 and followed up until they reached 3 years of age, 42.0% of hospital admissions were due to RTIs [6].

Despite these findings, data on the comparative frequency of RTIs in children with and without DS are lacking. Uncertainty remains around the burden of RTIs on children with DS in primary care, which has not been studied in detail until now. Families and carers are also lacking vital information regarding the relative risk of re-consultation, and the relative risk of consultation in the presence of certain comorbidities. This study aims to address the evidence gap by undertaking a retrospective cohort study of RTI-related healthcare utilisation in children with DS compared to controls.

## Methods

### Objectives

To quantify healthcare utilisation attributable to RTIs in children with and without DS from 1997 to 2010; to ascertain which children, with and without DS, are most at risk of increased RTI-related healthcare utilisation.

### Data sources and definitions

CALIBER is a database of linked routinely collected electronic health records (EHR) from England [7], comprising data from primary care (Clinical Practice Research Datalink, CPRD) [7], hospital admissions (Hospital Episode Statistics, HES) [7, 8], the Myocardial Ischaemia National Audit Project (MINAP) [9] and the national death registry at the Office for National Statistics (ONS). Read codes assigned by GPs to consultations, or ICD-10 codes assigned to hospitalisations, are used to ascertain the nature of healthcare utilisation.

We developed an algorithm that searched symptom and diagnosis codes, based on reviewing previous code lists used in UK databases for RTIs, and diagnostic and symptom codes were searched through the Read and ICD-10 dictionaries using the R CALIBERcode package [10]. Codes were classified as either “URTI”, “LRTI” or “Unclassified RTI” (i.e. uncertainty on whether it was a URTI or LRTI) based on previous code lists and after a consensus meeting including AS, ML, AH and LM. A similar process was undertaken to phenotype DS and comorbidities using any of the Read codes for DS in CPRD and ICD-10 codes for DS in HES. The code lists are available in Online Resource 1.

Within CALIBER, consultation and prescription rates were sourced from CPRD, and hospitalisation rates were sourced from HES. Rates were computed by dividing the number of episodes during the active period in the database by the total number of active person years. Each consultation for an RTI was followed up for up to 28 days or the first hospitalisation for an RTI within 28 days. RTIs were categorised following a ranking system based on RTI-type (LRTI > URTI > unclassified) and setting (secondary > primary care) if multiple episodes occurred on the same day.

We distinguished four different age groups based on author consensus: infants (0 to 1-year old), toddlers (1–5 years old), juniors (5–10 years old) and young persons (10–18 years old). For admissions lasting greater than 1 day, the length of stay was calculated as “length of stay = discharge date – admission date”. For admissions occurring over 1 day, the length of stay was the “length of stay = discharge date – admission date + 1 day”.

### Participants

We included all adults and children with DS identified in any of the CALIBER data sources between 1st January 1997 to 25th March 2010 by searching for any of the Read codes for DS in CPRD and by ICD-10 codes in HES recorded as either the primary or secondary discharge diagnosis (*n* = 3200). Individuals with an exit date from the database prior to their entry date to the database (i.e. patient records with data quality issues) were removed (*n* = 324). For each of the remaining adults and children with DS (*n* = 2876), five controls were frequency matched by GP, gender, birth year (± 5 years) and starting date of follow-up. Those who were more than 18 years old at the entry date were subsequently excluded from the study (*n* = 1884).

### Sample size and statistical models

Based on an Australian study of hospitalisations that noted an average of 0.8 and 0.1 RTI-attributable hospital admissions in individuals with and without DS, respectively [5], to identify a difference in hospitalisation rates as large as this between the two groups at 80% power using a 5% significance level, 20 individuals per group with the hospitalisation rates above would be required.

Poisson regression was used to calculate consultation, hospitalisation and prescription rates and rate ratios, and their corresponding 95% confidence intervals. Wald test was used to compare risk of admission following consultation. Due to highly skewed distributions, means and medians were reported, and the Wilcoxon rank–sum test was used to compare length of stay by RTI type, and time-to-hospitalisation.

All data management and analyses were performed using STATA statistical software version 13 and R version 3.2.3 via the UCL Data Safe Haven.

### Study registration

The protocol for this study was approved by the CPRD-independent scientific advisory committee, reference number 15_041R. The CALIBER record linkage has separate ethical approval (09/H0810/16) for observational clinical research.

## Results

### Cohort size, demographics and comorbidities

This study identified 992 children with DS. They were followed up for a total of 4681 person years at risk, a mean of 4.72 years per child. The 4874 controls were followed up for a total of 22,837 person years at risk, a mean of 4.69 years per child. Table 1 displays the demographics and comorbidities of the study populations.

**Table 1:** Demographics and comorbidities of the study population

	Children with DS	Control
Patient	992 (100.0%)	4874 (100.0%)
Gender		
Male	528 (53.2%)	2626 (53.9%)
Female	464 (46.8%)	2248 (46.1%)
Age at entry into cohort		
Infants (0–1 year)	252 (25.4%)	1247 (25.6%)
Toddlers (1–5 years)	224 (22.6%)	1133 (23.2%)
Juniors (5–10 years)	208 (21.0%)	1044 (21.3%)
Young persons (10–18 years)	308 (29.8%)	1454 (29.8%)
Ethnicity		
Asian or Asian British	56 (3.3%)	211 (2.5%)
Black or Black British	48 (2.8%)	189 (2.4%)
Chinese or ‘Other’ Group	30 (1.7%)	114 (1.35%)
Mixed	72 (4.2%)	393 (4.7%)
Unknown	504 (29.5%)	4005 (47.5%)
White	1001 (58.5%)	3523 (41.8%)
Comorbidities		
Asthma	136 (13.7%)	618 (12.7%)
CHD	393 (39.6%)	48 (1.0%)
Diabetes	11 (1.1%)	20 (0.4%)
Epilepsy	18 (1.8%)	34 (0.7%)
Hypothyroidism	103 (10.4%)	11 (0.2%)

### Consultation and hospitalisation rates

RTI-related healthcare utilisation for children with DS is high, and higher than for controls. RTI-related GP consultation rates were 64 per 100 person years for children with DS, and 36 per 100 person years for controls. Corresponding RTI-related hospitalisation rates were 7 per 100 person years for children with DS, and 1 per 100 person years for controls (see Table 2).

**Table 2:** RTI-related GP consultation (top) and hospitalisation (bottom) rates by RTI type in children with DS and controls

Classification	Children with DS		Controls		Children with DS vs controls		
	# of episodes	Rate per person year [95% CI]	# of episodes	Rate per person year [95% CI]	RR [95% CI]	Adjusted RR [95% CI]	*p* value
RTI-related GP consultation rates							
All	6013	0.638 [0.582–0.699]	13,957	0.363 [0.348–0.378]	1.760 [1.647–1.880]	1.726 [1.617–1.843]	< 0.0001
URTI	3442	0.421 [0.385–0.460]	9093	0.258 [0.247–0.270]	1.628 [1.514–1.750]	1.604 [1.493–1.723]	< 0.0001
LRTI	874	0.119 [0.107–0.134]	1039	0.034 [0.031–0.037]	3.508 [3.108–3.955]	3.589 [3.188–4.041]	< 0.0001
Unclassified RTI	1697	0.199 [0.179–0.220]	3825	0.114 [0.109–0.120]	1.739 [1.588–1.902]	1.759 [1.609–1.923]	< 0.0001
RTI-related hospitalisation rates							
All	473	0.067 [0.058–0.077]	327	0.013 [0.011–0.014]	5.342 [4.506–6.332]	5.693 [4.818–6.727]	< 0.0001
URTI	205	0.035 [0.030–0.043]	187	0.007 [0.006–0.009]	4.814 [3.842–6.029]	4.989 [4.007–6.211]	< 0.0001
LRTI	213	0.032 [0.026–0.038]	73	0.003 [0.002–0.004]	10.557 [7.847–14.321]	11.295 [8.448–15.101]	< 0.0001
Unclassified RTI	55	0.010 [0.007–0.013]	67	0.003 [0.002–0.004]	3.468 [2.314–5.158]	3.578 [2.444–5.239]	< 0.0001

When adjusted for age group, children with DS are nearly twice as likely (adjusted relative risk (RR) 1.73; 95% confidence interval (CI) 1.62–1.84) as matched controls to present to their GP with an RTI in general, and six times more likely (adjusted RR 5.69; 95% CI 4.82–6.73) to be admitted to hospital with an RTI (see Table 2).

Overall, 74.1% and 73.4% of RTI-related hospitalisations occur without any prior GP consultation in for children with DS and controls, respectively.

Approximately 2% of children with DS are admitted to hospital in the 28 days following a GP consultation for RTI compared to 0.7% of controls [RR 3.15 (95% CI 2.35–4.24), unadjusted] (Table 3).

**Table 3:** Risk of RTI-related hospitalisation following an RTI-related GP consultation within 28 days in children with DS and controls

Classification	Children with DS			Controls			Children with DS vs Controls	
	# of consultations	# of hospitalisations	Risk [95% CI]	# of consultations	# of hospitalisations	Risk [95% CI]	Risk ratio [95% CI]	*p* value
All	4685	97	0.021 [0.017–0.025]	11,877	78	0.007 [0.005–0.008]	3.153 [2.345–4.239]	< 0.0001
URTI	2769	42	0.015 [0.011–0.021]	7915	43	0.005 [0.004–0.007]	2.792 [1.829–4.262]	< 0.0001
LRTI	621	15	0.024 [0.014–0.040]	838	7	0.008 [0.003–0.017]	2.892 [1.186–7.050]	0.0168
Unclassified RTI	1295	40	0.031 [0.022–0.042]	3124	28	0.009 [0.006–0.013]	3.446 [2.135–5.561]	< 0.0001

Time to RTI-related hospitalisation following an RTI-related GP consultation is the same in both groups, with a median of 8.0 days (95% CI 3.0–19.0) in children with DS and 8.0 days (95% CI 2.0–18.0) in matched controls. However, children with DS are more likely (OR 1.69; 95% CI 1.57–1.82) to re-consult with their GP for an RTI within 28 days of a prior RTI-related consultation compared to matched controls, with 24.3% children with DS re-consulting compared to 16.0% of matched controls.

**Fig. 1:**
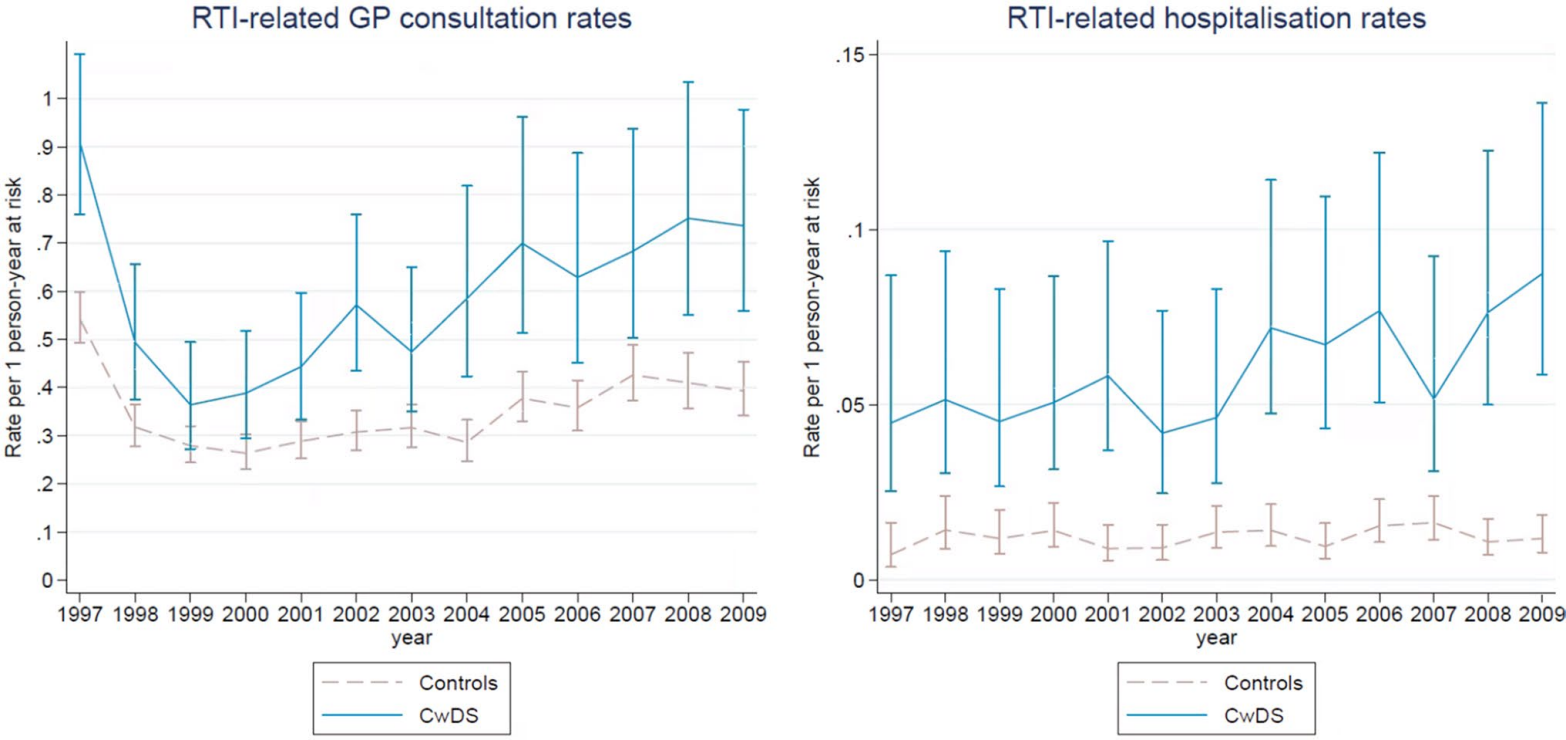
Annual RTI-related GP consultation and hospitalisation rates in children with DS compared to controls

Over the study period between 1997 and 2010, RTI-related GP consultation and hospitalisation rates were consistently higher in children with DS compared to controls. The disparities increased over time; in 1999, children with DS had a third more RTI-related consultations, compared to 80% more in 2009. An increase was similarly noted for RTI-related hospitalisations. This is shown in Fig. 1.

### Healthcare utilisation by type of RTI

Across all RTI types (i.e. LRTI, URTI, and unclassified RTI), children with DS consistently consult GPs and are hospitalised for RTIs more frequently compared to controls. The differences between children with DS and controls were most pronounced for LRTIs in GP consultations (RR 3.59; 95% CI 3.19–4.04) and hospitalisations (RR 11.30; 95% CI 8.45–15.10). This is illustrated in Table 2.

### Length of hospitalisations for RTIs

Overall, as shown in Table 4, the length of stay in hospital due to RTIs is significantly longer for children with DS (mean 5.2; 95% CI 5.0–5.4 days per admission) compared to controls (mean 2.4; 95% CI 2.2–2.6, *p* < 0.0001). In keeping with LRTIs being more severe than URTIs or unclassified RTIs, LRTI-related hospitalisations last longer for children with DS (mean 7.8; 95% CI 7.4–8.1 days per admission) and controls (mean 4.2; 95% CI 3.8–4.7).

**Table 4:** Length of stay in hospital for LRTI, URTI and unclassified RTI in days in children with DS and controls

Classification	Children with DS		Controls		*p* value
	Mean [95% CI]	Median [IQR]	Mean [95% CI]	Median [IQR]	
All	5.2 [5.0–5.4]	2.0 [1.0–5.0]	2.4 [2.2–2.6]	2.0 [1.0–2.0]	< 0.0001
URTI	2.4 [2.2–2.6]	2.0 [1.0–2.0]	1.9 [1.7–2.1]	1.0 [1.0–2.0]	0.0325
LRTI	7.8 [7.4–8.1]	5.0 [3.0–10.0]	4.2 [3.8–4.7]	3.0 [2.0–5.0]	< 0.0001
Unclassified RTI	5.6 [5.0–6.3]	2.0 [1.0–4.0]	1.9 [1.6–2.3]	1.0 [1.0–2.0]	0.0265

### Healthcare utilisation stratified by age, gender and comorbidities

Consultation and hospitalisation rates for RTIs were higher in children with DS compared to controls across all age groups. This difference was particularly pronounced for hospitalisations (see Table 5).

**Table 5:** RTI-related GP consultation (top) and hospitalisation (bottom) rates stratified by age groups and compared between children with DS and controls

Classification	Children with DS		Controls		Children with DS vs controls	
	# of episodes	Rate per person year [95% CI]	# of episodes	Rate per person year [95% CI]	RR [95% CI]	*p* value
RTI-related GP consultation rates						
Infants	270	1.495 [1.252–1.790]	961	1.236 [1.140–1.341]	1.210 [0.975–1.490]	0.0371
Toddlers	2387	1.225 [1.052–1.422]	6185	0.813 [0.761–0.867]	1.508 [1.339–1.695]	< 0.0001
Juniors	1727	0.715 [0.621–0.822]	3365	0.324 [0.302–0.346]	2.211 [1.946–2.505]	< 0.0001
Young person	1629	0.364 [0.319–0.414]	3446	0.195 [0.184–0.208]	1.861 [1.648–2.096]	< 0.0001
RTI-related hospitalisation rates						
Infants	73	0.515 [0.388–0.695]	56	0.079 [0.059–0.109]	6.491 [4.190–10.087]	< 0.0001
Toddlers	240	0.140 [0.116–0.171]	176	0.031 [0.026–0.036]	4.582 [3.554–5.893]	< 0.0001
Juniors	93	0.058 [0.046–0.075]	56	0.008 [0.006–0.011]	7.330 [5.002–10.792]	< 0.0001
Young person	67	0.024 [0.018–0.032]	39	0.004 [0.003–0.005]	6.669 [4.296–10.432]	< 0.0001

There is no significant gender-based difference for RTI-related GP consultation rates for children with DS (adjusted RR 1.08; 95% CI 0.96–1.21) and matched controls (adjusted RR 0.99; 95% CI 0.93–1.05). Girls have lower RTI-related hospitalisation rates compared to boys in both children with DS (adjusted RR 0.70; 95% CI 0.55–0.89) and matched controls (adjusted RR 0.69; 95% CI 0.54–0.88).

Amongst children with DS, those who have congenital heart disease (CHD) have increased RTI-related consultation (adjusted RR 1.21; 95% CI 1.04–1.40) and hospitalisation rates (adjusted RR 3.07; 95% CI 2.38–3.95) compared to those without CHD. A similar pattern is observed in controls for RTI-related consultations (adjusted RR 1.63; 95% 1.00–2.67) and hospitalisations (adjusted RR 3.89; 95% CI 1.25–12.11).

Children with DS with asthma attend their GP for RTI-related consultations more often compared to children with DS without asthma (adjusted RR 2.06; 95% CI 1.74–2.44) and are hospitalised more often (adjusted RR 1.68; 95% CI 1.23–2.30). Controls with asthma attend their GP more often (adjusted RR 1.75; 95% CI 1.61–1.91) and are hospitalised more often (adjusted RR 2.65; 95% CI 2.01–3.50) for RTIs relative to controls without asthma.

### Antibiotic prescribing

Children with DS are prescribed twice as many antibiotics compared to matched controls, with a rate of 77 per 100 person years, compared to 32 per 100 person years for controls (adjusted RR 2.34; 95% CI 2.19–2.49). When restricted to antibiotic prescriptions prescribed on the same day as an RTI-related GP consultation, children with DS are twice as likely to be prescribed an antibiotic for an RTI compared to matched controls, with a rate of 42 per 100 person years for children with DS compared to 19 per 100 person years for controls (adjusted RR 2.26; 95% CI 2.10–2.43).

When stratified by RTI type, children with DS receive significantly more antibiotics on the same day as an RTI-related GP consultations when presenting with URTIs (57.0%; 95% CI 55.3–58.6% vs 47.9%; 95% CI 46.9–49.0%) and unclassified RTIs (52.9%; 95% CI 50.5–55.3% vs 32.0%; 95% CI 30.5–33.5%). There is no significant difference in the high proportion of LRTIs that are prescribed antibiotics in children with DS compared with controls (87.0% vs. 82.5%).

When stratified by age group, infants with DS have the highest rate of RTI-related antibiotic prescribing compared to all other groups including controls, with rates of 80 per 100 person years (95% CI 65–99). Prescribing rates are significantly higher for each age group in DS compared to the equivalent age group amongst controls.

## Discussion

This study demonstrates that RTIs in children with DS lead to a significant number of presentations to GPs and to hospitals every year, posing a significant burden on patients and their families. Compared to controls, children with DS attend GP consultations for RTIs almost twice as often, are hospitalised six times as often, stay in hospital longer, and are prescribed antibiotics more frequently. The presence of comorbidities increased RTI-related healthcare utilisation for both groups. Children with DS and controls were hospitalised and attended GPs for RTIs less often as they aged.

Children with DS were three times more likely to be admitted to hospital for an RTI following an RTI-related GP consultation than controls, with a risk of 2.1%, compared to 0.7% amongst controls. The finding of 0.7% in controls (95% CI 0.5–0.8%) is similar to a recent UK population based cohort study which noted a baseline risk of 0.9% (95% CI 0.7–1.2%) [11].

Notably, for both children with DS and controls, more than 70% of RTI-related hospitalisations were not preceded by a recorded GP consultation.

There are number of limitations to our study, described below.

## Strengths and limitations

This is the largest study of RTI-related healthcare utilisation in children with DS worldwide (*n* = 992) and provides novel insights as the first study known to us to assess healthcare utilisation and antibiotic prescribing in primary care for children with DS, as well as to compare them with controls. It provides valuable data on the prevalence of antibiotic prescribing, and the associated impact of key comorbidities. Comorbidity prevalence figures in this study are similar to those in existing literature. The calculated prevalence of CHD in DS of 39.6% compares to rates of 33.7–58.2% in other studies [12–15]. A prevalence of 13.7% for asthma compares to rates of 3.1–19.4% [16, 17].

There are number of limitations to this study which should be noted when considering our findings. By relying on READ and ICD-10 codes for diagnoses, this study may be subject to misclassification bias. It is known that considerable inter-practice variation exists in coding certain conditions such as RTIs. For example, READ or ICD-10 codes for “respiratory tract infection” could be either an URTI or LRTI. We aimed to address this by separately considering unclassified RTI types, but misclassification bias may remain. Misclassification may particularly be present for asthma, which is not typically diagnosed in the under 5s in the UK. Individual episodes could have been missed through non-recording by GPs, or use of free text entries, although it is unknown whether this would differ between children with DS and controls. An additional proportion of medically attended RTIs in both children with DS and controls will be missed, as RTIs are seen not only by GPs but also at other ambulatory care centres (i.e. urgent care centres, out-of-hours GP) and A&E. Finally, and importantly, research has noted that most RTIs do not lead to a GP consultation [18]. There is little qualitative evidence on healthcare seeking behaviour in families of children with DS, and whether this varies from other patient groups. Until such evidence can be gathered, we cannot know whether the data partially reflects a difference in propensity to consult when children with DS and controls have RTIs, rather than a true difference in RTI incidence.

### Implications for clinical practice and research

Healthcare professionals in primary care should be vigilant when assessing children with DS with the knowledge that subsequent hospital admissions are more likely. Morphological and functional anomalies of the airway causing (partial) obstruction, particularly midfacial hypoplasia and airway malacia [19], alongside immunological variations including reduced leukocytes, particularly T- and B-cell subpopulations [20–23], increase susceptibility to and severity of respiratory infections. Increased admissions may also be due to uncertainty regarding speed of deterioration or oxygen requirements, and many admissions may be precautionary, also reflected by the higher prescribing rates in children with DS. In this context, parents have reported that some doctors seem ‘afraid’ of children with DS [24]. It will be important to identify whether the high rates of admission are related to severity of the infection, time at presentation, time and type of antibiotics prescribed, or other factors. Further research should determine why more than 70% of RTI-related hospitalisations were not preceded by a recorded GP consultation as this may represent an opportunity to avert admission through timely community-based treatment. Regional analyses may be helpful, comparing Clinical Commissioning Groups with high and low rates of RTI-related health utilisation, to highlight areas of best practice and whether local community initiatives exist that can avert unnecessary attendances.

This study also found that certain subgroups are at greater risk of hospitalisation. It may be that those at risk of RTI-related hospitalisation could be defined in greater detail, thus enabling the development of a symptom-based scoring algorithm modelled on algorithms that already exist for many other conditions [11, 25, 26]. However, symptoms are not well recorded in routine records. Although not explored by this study, further research may also want to examine prescribing rates for antivirals for influenza in children with DS. These are rarely prescribed for children without DS, despite recommendations to do so [27, 28]. Regarding public health recommendations, this study lends weight to the recommendation by the Down’s Syndrome Medical Interest Group that children with DS should be considered for annual influenza vaccination at all ages [29], particularly in infancy.

## Conclusion

This is the first study of RTI-related healthcare utilisation in children with DS compared to controls utilising linked primary and secondary care data. We show that healthcare utilisation is high in this population and higher than controls. Further research is recommended, carefully quantifying healthcare behaviours and health professional decision-making, to optimise care for this at-risk group.

## Electronic supplementary material

Below is the link to the electronic supplementary material.

Supplementary file1 (PDF 955 kb)
