# Correction to: Respiratory tract infection-related healthcare utilisation in children with Down’s syndrome

**DOI:** 10.1007/s15010-020-01425-4

**Published:** 2020-04-27

**Authors:** Logan Manikam, Anne G. M. Schilder, Monica Lakhanpaul, Peter Littlejohns, Emma C. Alexander, Andrew Hayward

**Affiliations:** 1 UCL Institute of Epidemiology and Healthcare, University College London, 1-19 Torrington Place, London, WC1E 6BT UK; 2 UCL Institute of Health Informatics Research, University College London, 222 Euston Road, London, NW1 2DA UK; 3 National Institute of Health Research University College London Hospitals Biomedical Research Centre, 149 Tottenham Court Road, London, W1T 7DN UK; 4 evidENT, UCL Ear Institute, University College London, 332 Grays Inn Road, London, WC1X 8DA UK; 5 Population, Policy & Practice, UCL Great Ormond Street Institute of Child Health, University College London, 30 Guilford Street, London, WC1N 1EH UK; 6 Whittington Health NHS Trust, Magdala Avenue, London, N19 5NF UK; 7 Centre for Implementation Science, Institute of Psychiatry, Psychology and Neurosciences, King’s College London, 6 De Crespigny Park, Camberwell, London, SE5 8AB UK; 8 Paediatric Liver, GI and Nutrition Centre and Mowatlabs, King’s College Hospital, Denmark Hill, London, SE5 9RS UK

## Correction to: Infection 10.1007/s15010-020-01408-5

The original version of this article unfortunately contained an omission.

The Acknowledgements section was missing. The Acknowledgements are now given below.

