# Supplementary material for: Respiratory tract infection-related healthcare utilisation in children with Down’s syndrome
Source: Infection. 2020 Mar 14;48(3):403–10. doi: 10.1007/s15010-020-01408-5 (PMC7095390; doi:10.1007/s15010-020-01408-5)
Supplement: Supplementary file 1 — Supplementary file1 (PDF 955 kb) [file 15010_2020_1408_MOESM1_ESM.pdf]

SUPPLEMENTARY MATERIAL: CODE LISTS

Respiratory Tract Infection Related Healthcare Utilisation in Children with Down's Syndrome

Journal Name:

Infection

Author Names:

Logan Manikam[1,2]
Monica Lakhanpaul[3]
Anne Schilder[4]
Peter Littlejohns[5]
Emma Alexander[6]
Andrew Hayward[7]

Affililation:

[1] –Institute of Epidemiology and Healthcare, University College London,1-19 Torrington Place, London, WC1E 6BT, UK.
[2] – Institute of Health Informatics Research, University College London, 222 Euston Road, London, NW1 2DA, UK.
[3] – National Institute of Health Research University College London Hospitals Biomedical Research Centre, 149 Tottenham Court Road, London, W1T 7DN, UK.
[4] - evidENT, UCL Ear Institute, 332 Grays Inn Road, London, WC1X 8DA, UK.
[5] – Population, Policy & Practice, Great Ormond Street Institute of Child Health, University College London, 30 Guilford Street, London, WC1N 1EH, UK.
[6] – Centre for Implementation Science, Institute of Psychiatry, Psychology and Neurosciences, King's College London, 6 De Crespigny Park, Camberwell, London, SE5 8AB, UK.
[7] – Paediatric Liver, GI and Nutrition Centre and Mowatlabs, King's College Hospital, Denmark Hill, London, SE5 9RS, UK.

Corresponding Author:

Dr Logan Manikam, logan.manikam.10@alumni.ucl.ac.uk, UCL Institute of Epidemiology and Healthcare, 1-19 Torrington Place, London, WC1E 6BT, UK.

Table A 1. Condition and co-morbidities searched using the RCALIBERcodelists package

Sub-Category	Conditions Searched	Conditions Not Searched	Codes Searched
**Conditions Associated with Breathing Difficulties in Down's Syndrome**			
Respiratory	Obstructive sleep apnoea		Sleep|Apnoe*
	Chronic lung disease		Lung
	Tracheostomy		Trache*
		Subpleural cysts	
Cardiovascular	Congenital heart disease		Congenital|Heart
	Atrioventricular canal defect		Atri*
	Atrial & ventricular septal defects		Atri*|Ventric*
	Aortic regurgitation		Regurg*
	Patent ductus arteriosus		Duct*
	Tetralogy of Fallot		Fallot
	Double outlet right ventricle		Ventric*
	Mitral valve prolapse		Valve
	Acquired valve disease		Valve
Gastrointestinal	Gastroesophageal reflux disease		Reflux*
	Swallowing dysfunction		Swallow*
	Oesophageal atresia repair		Oesophag*
	Tracheo-oesophageal fistula		Trache*
Anatomical	Tracheal bronchus		Trache*
	Tracheomalacia		Trache*
		Small jaw	
		Macroglossia	
		Narrow nasopharynx	
		Adenotonsillar hypertrophy	
		Choanal stenosis	
		Laryngomalacia	
		Narrow trachea	
		Mid-face hypoplasia	
		Subglottic stenosis	
		Small upper airway	
Contributory factors		Obesity	
		Hypotonia	
**Conditions Associated with Infections in Down's Syndrome**			
Cancer	Acute Leukaemia		Acute|Leukaemi*
	Myeloproliferative disease		Myeloproliferative
Misc		Increased mucus secretions	
		Reduced ciliary beat frequency	
		B-cell function abnormality	
		Decreased neutrophil chemotaxis	
		Thymic abnormalities	
		Alteration of levels of immunoglobulin subclasses	
		Alterations in response to vaccinations	
		Reduced T and B lymphocyte subpopulations	

		Ciliary dysfunction	
**Population: Down's Syndrome**			
Down's Syndrome	Down's Syndrome		Down|Trisomy|Mongol
**Infections**			
URTI	Common Cold		Common|upper|infection
	Rhinitis		Acute|rhinitis
	Sinusitis		Sinusit*|rhinosinusitis
		Nasopharyngitis	
		Pharyngitis	
	Epiglottits		Epiglot*
		Laryngitis	
		Laryngotracheitis	
	Tracheitis		Trache*
		Tonsillitis	
		Otitis Media	
LRTI	Chest infection		Chest|Lower resp*
	Bronchitis		Bronchi*
	Bronchiolitis		Bronchi*
	Croup |Laryngotracheobronchitis		Croup |Laryngotracheo
	Pneumonia		Pneumon*
SBIs	Meningitis		Mening*
	Septicaemia		Sepsis |Septic*
	Endocarditis		Endocard*
	Abscess		Abscess
Other Infections	Skin and soft tissue		Infect*
	Urinary		Infect*
	Gastro		Infect*
	TB (All subtypes)		Infect*| Lung
	Named organism infections w/out site		Infect*
	Otitis externa		Infect*
	Bone		Infect*

Table A 2. List of Read codes

Down's Syndrome		
	Q909	Down's Syndrome, unspecified
	1543	Down's Syndrome- trisomy 21

10759		Down’s Syndrome NOS
19062		Partial Trisomy Syndromes
18415		Trisomy 21
Q929		Trisomy and partial trisomy of autosomes, unspecified
Q901		Trisomy 21, mosaicism (mitotic nondisjunction)
Q900		Trisomy 21, meiotic nondisjunction

LRTI

22448	POSSIBLE	O/E - intercostal recession
25722	POSSIBLE	O/E - subcostal recession
7092	POSSIBLE	Recurrent wheezy bronchitis
152	POSSIBLE	Wheezy bronchitis
8582	POSSIBLE	O/E - chest findings
5978	PROBABLE	Acute wheezy bronchitis
13573	PROBABLE	Influenza with bronchopneumonia
8539	POSSIBLE	O/E - shallow breathing
14976	PROBABLE	Viral pneumonia NOS
5202	PROBABLE	Viral pneumonia
44425	PROBABLE	Pleural empyema
7000	POSSIBLE	O/E - dyspnoea
1934	PROBABLE	Laryngotracheobronchitis
18451	PROBABLE	Acute bronchiolitis due to respiratory syncytial virus
17359	PROBABLE	Chest infection - unspecified bronchitis
10321	POSSIBLE	O/E - consolidation
41137	PROBABLE	Acute bronchitis or bronchiolitis NOS
8318	PROBABLE	Lung consolidation
22795	PROBABLE	Chest infection - other bacterial pneumonia
3683	PROBABLE	Basal pneumonia due to unspecified organism
23095	PROBABLE	Bacterial pneumonia NOS
25694	PROBABLE	Pneumonia due to other specified organisms
1576	PROBABLE	Pneumonia due to mycoplasma pneumoniae
3480	PROBABLE	Bronchitis NOS
3163	PROBABLE	Tracheobronchitis NOS
10114	POSSIBLE	O/E - tachypnoea
9639	PROBABLE	Lobar pneumonia due to unspecified organism
5324	PROBABLE	Atypical pneumonia

	25571	POSSIBLE	O/E - coarse crepitations
	1382	PROBABLE	Acute viral bronchitis unspecified
	10086	PROBABLE	Pneumonia and influenza
	11101	PROBABLE	Acute tracheobronchitis
	9062	POSSIBLE	O/E - crepitations
	4626	POSSIBLE	O/E - rhonchi present
	29669	PROBABLE	Acute bronchitis and bronchiolitis
	1849	PROBABLE	Lobar (pneumococcal) pneumonia
	6094	PROBABLE	Pneumonia or influenza NOS
	37447	PROBABLE	Acute lower respiratory tract infection
	886	PROBABLE	Bronchopneumonia due to unspecified organism
	4899	PROBABLE	Recurrent chest infection
	978	PROBABLE	Pleurisy
	572	PROBABLE	Pneumonia due to unspecified organism
	148	PROBABLE	Bronchitis unspecified
	6124	PROBABLE	Acute lower respiratory tract infection
	1019	PROBABLE	Acute bronchiolitis
	3358	PROBABLE	Lower resp tract infection
	312	PROBABLE	Acute bronchitis
	2581	PROBABLE	Chest infection NOS
	68	PROBABLE	Chest infection
	2375	PROBABLE	Empyema
RTI			
	6475	POSSIBLE	[D]Respiratory system and chest symptoms
	7118	POSSIBLE	O/E - pyrexia - ? cause
	5892	POSSIBLE	O/E - fever
	16660	POSSIBLE	O/E - nose crusting
	4519	POSSIBLE	H/O: bronchitis
	1740	POSSIBLE	Night sweats
	2389	POSSIBLE	[D]Pyrexia of unknown origin
	6086	POSSIBLE	Pyrexia symptoms
	6484	POSSIBLE	Temperature symptoms
	6065	POSSIBLE	Fever symptoms
	5859	POSSIBLE	Feels hot/feverish
	1020	POSSIBLE	[D]Fever NOS

24181	POSSIBLE	Sputum: mucopurulent
15430	POSSIBLE	[D]Sputum abnormal - colour
14804	POSSIBLE	Sputum appears infected
1025	POSSIBLE	Bronchial cough
735	POSSIBLE	[D]Breathlessness
7707	POSSIBLE	Cough symptom NOS
21113	PROBABLE	Acute respiratory infection NOS
9807	POSSIBLE	Sputum - symptom
7708	POSSIBLE	Productive cough-yellow sputum
550	POSSIBLE	Rhinorrhoea
7074	PROBABLE	Respiratory infection NOS
5896	POSSIBLE	Dyspnoea - symptom
5175	POSSIBLE	Breathlessness symptom
7773	POSSIBLE	Productive cough -green sputum
2931	POSSIBLE	Difficulty breathing
3628	POSSIBLE	Persistent cough
1234	POSSIBLE	Productive cough NOS
4931	POSSIBLE	Dry cough
1429	POSSIBLE	Breathlessness
1160	POSSIBLE	[D]Cough
4822	POSSIBLE	Shortness of breath
293	PROBABLE	Respiratory tract infection
292	POSSIBLE	Chesty cough
1273	POSSIBLE	C/O - cough
92	POSSIBLE	Cough

URTI

32802	POSSIBLE	Other upper respiratory tract diseases
2097	PROBABLE	Nasal cavity and sinus disease NOS
16986	POSSIBLE	Nose running
8975	POSSIBLE	Catarrh unspecified
11139	POSSIBLE	Blocked nose
1309	PROBABLE	Nasal infection
9483	POSSIBLE	Sinus congestion
1401	POSSIBLE	Nasal obstruction

7479	POSSIBLE	Catarrh - eustachian
3821	POSSIBLE	Rhinitis - acute
3110	POSSIBLE	Nasal congestion
6481	POSSIBLE	Nasal symptoms
5765	POSSIBLE	C/O - catarrh
1513	PROBABLE	Infection ear
5577	PROBABLE	Nonsuppurative otitis media + eustachian tube disorders
731	POSSIBLE	Otalgia
5813	POSSIBLE	Earache symptoms
10781	PROBABLE	Acute suppurative otitis media tympanic membrane intact
15774	PROBABLE	Influenza with laryngitis
16184	PROBABLE	Streptococcal sore throat with scarlatina NOS
16147	POSSIBLE	O/E - purulent ear discharge
22131	POSSIBLE	O/E - tonsils hyperaemic
20372	PROBABLE	Acute suppurative otitis media NOS
97279	PROBABLE	[X]Influenza+other manifestations, virus not identified
21012	PROBABLE	Acute mucoid otitis media
20669	PROBABLE	Acute suppurative otitis media tympanic membrane ruptured
10641	PROBABLE	Acute epiglottitis (non strep)
17899	PROBABLE	Acute bacterial pharyngitis
20618	POSSIBLE	O/E - nose discharge
7266	PROBABLE	O/E - follicular tonsillitis
14791	PROBABLE	Influenza with gastrointestinal tract involvement
4718	PROBABLE	Pharyngolaryngitis
8570	POSSIBLE	O/E - rhinorrhoea
3605	PROBABLE	Peritonsillar abscess - quinsy
10087	PROBABLE	Acute laryngotracheitis
9973	PROBABLE	Recurrent acute otitis media
15287	POSSIBLE	Sore throat symptom NOS
4902	PROBABLE	Streptococcal pharyngitis
6958	POSSIBLE	Otorrhagia
6620	PROBABLE	Febrile cold
6498	POSSIBLE	O/E - tonsils mod. enlarged
16388	PROBABLE	Influenza NOS
18363	POSSIBLE	O/E - tympanic membrane red

5115	PROBABLE	Acute viral laryngitis unspecified
5102	PROBABLE	Serous otitis media NOS
19431	PROBABLE	Croup
15410	POSSIBLE	Throat symptom NOS
10156	PROBABLE	Acute bacterial tonsillitis
18371	PROBABLE	Acute otitis media with effusion
3260	PROBABLE	Acute nasopharyngitis
4221	PROBABLE	Recurrent upper respiratory tract infection
26010	PROBABLE	Other acute upper respiratory infections
7021	PROBABLE	Acute maxillary sinusitis
20104	PROBABLE	Acute tonsillitis NOS
9357	PROBABLE	Acute viral tonsillitis
8950	PROBABLE	Feverish cold
20374	PROBABLE	Acute nonsuppurative otitis media NOS
1285	PROBABLE	Laryngotracheitis
2984	PROBABLE	Frontal sinusitis
2476	PROBABLE	Chest cold
5148	PROBABLE	Acute secretory otitis media
5947	PROBABLE	Influenza like illness
911	PROBABLE	Quinsy
1765	PROBABLE	Streptococcal sore throat
1747	PROBABLE	Recurrent acute tonsillitis
9093	PROBABLE	Pyrexial cold
3624	PROBABLE	Maxillary sinusitis
4061	PROBABLE	Acute follicular tonsillitis
8496	PROBABLE	Streptococcal tonsillitis
7730	PROBABLE	Acute serous otitis media
386	POSSIBLE	Throat pain
11499	PROBABLE	Throat infection - tonsillitis
5935	POSSIBLE	O/E - nasal discharge
6466	PROBABLE	Viral sore throat NOS
5390	PROBABLE	Catarrhal otitis media NOS
4868	PROBABLE	Acute viral pharyngitis
8980	POSSIBLE	Influenza-like symptoms
5553	POSSIBLE	Has a sore throat

14931	POSSIBLE	Inflamed throat
1134	PROBABLE	Acute bilateral otitis media
1390	POSSIBLE	Snuffles
6014	POSSIBLE	Sore throat NOS
5806	POSSIBLE	O/E - painful ear
407	PROBABLE	Acute pharyngitis NOS
556	PROBABLE	Influenza
896	PROBABLE	Nasal catarrh - acute
2137	PROBABLE	Acute suppurative otitis media
638	POSSIBLE	Otorrhoea
3694	PROBABLE	Acute left otitis media
4348	PROBABLE	Acute right otitis media
2125	PROBABLE	Tonsillitis
1257	PROBABLE	Acute tracheitis
6421	PROBABLE	Viral upper respiratory tract infection NOS
1142	PROBABLE	Croup
310	PROBABLE	Throat infection - pharyngitis
368	PROBABLE	Common cold
5887	PROBABLE	Acute non suppurative otitis media
1246	PROBABLE	Coryza - acute
142	PROBABLE	Acute laryngitis
1474	PROBABLE	Suppurative and unspecified otitis media
243	PROBABLE	Sinusitis
2157	PROBABLE	Flu like illness
893	PROBABLE	Acute pharyngitis
6294	PROBABLE	Acute upper respiratory tract infection
980	PROBABLE	Acute sinusitis
267	PROBABLE	Otitis media NOS
404	POSSIBLE	Throat soreness
5755	POSSIBLE	Sore throat symptom
138	PROBABLE	Acute tonsillitis
2637	PROBABLE	Upper respiratory tract infection NOS
76	PROBABLE	Upper respiratory infection NOS

asthma

39570	Asthma causes night symptoms 1 to 2 times per month
38145	Asthma limits walking on the flat
13175	Asthma disturbs sleep frequently
11370	Asthma confirmed
1555	Bronchial asthma
7191	Asthma limiting activities
3018	Mild asthma
4442	Asthma unspecified
13065	Moderate asthma
24884	Asthma causes daytime symptoms 1 to 2 times per week
7416	Asthma disturbing sleep
31225	Asthma causes daytime symptoms 1 to 2 times per month
13066	Asthma - currently dormant
233	Severe asthma attack
26504	Asthma never restricts exercise
16655	Asthma monitoring admin.
19520	Asthma treatment compliance satisfactory
232	Asthma attack
38143	Asthma never disturbs sleep
10274	Asthma medication review
19539	Asthma monitoring check done
5515	Seen in asthma clinic
13064	Asthma severity
7378	Asthma management plan given
185	Acute exacerbation of asthma
25707	Asthma monitor 1st letter
13176	Asthma follow-up
10043	Asthma annual review
81	Asthma monitoring
78	Asthma

cardiac

8464	Acute cor pulmonale
39885	Revision of closure of defect of atrioventricular septum
50626	Close defect interventricular septum using pericardial patch
12889	Percut transluminal prosth occlusion patent ductus arterios

12312	Pulmonary valve disorders
5058	Mitral incompetence, non-rheumatic
49551	Tricuspid stenosis and regurgitation, cause unspecified
3810	Complete atrioventricular block
35372	Tricuspid regurgitation, non-rheumatic
41569	Other specified catheterisation of heart
3169	Heart valve and adjacent structures operations NOS
36668	Closure of patent ductus arteriosus NEC
42132	Ventricular septal defect, unspecified
48207	Other operations on heart
54772	Other specified ventricular septal defect
89256	Congenital heart disease
30443	Mitral valve disease NOS
12844	Repair of tetralogy of Fallot
48331	Primary closure of defect of atrioventricular septum NEC
4939	Bacterial endocarditis
20940	Repair of subaortic stenosis
19188	Percut translum prosth occlus patent ductus arteriosus (PDA)
63046	Tetralogy of Fallot NOS
1267	Mitral valve diseases
30725	Aortic arch anomalies
84407	Other operations on ventricles of heart
34902	Closure of defect of interatrial septum
10078	Diseases of mitral and aortic valves
1007	Aortic incompetence alone, cause unspecified
48205	Congenital malforms of cardiac chambers+connections unsp
6886	Congenital aortic valve stenosis
39810	Heart wall, septum and chamber operations
3301	Coarctation of aorta
73602	Repair of atrium NEC
16191	Closure of defect of interatrial septum NOS
9573	Ligation of patent ductus arteriosus
3300	Bicuspid aortic valve
16539	Subaortic stenosis
2977	Mitral valve incompetence

30173	Valves of heart and adjacent structures operations
3863	Cyanotic congenital heart disease NOS
12775	Acute and subacute endocarditis
15133	Replacement of aortic valve NEC
17088	H/O: cardiac surgery
10079	Right heart failure
11878	Mitral and aortic regurgitation
5621	Other congenital heart anomalies
30340	Closure of defect of unspecified septum of heart
17328	Aorta operations
38436	Closure of defect of atrioventricular septum NOS
36575	Closure of defect of interventricular septum
19019	Aortic valve disorders NOS
18820	H/O: cardiac anomaly
7342	Open correction of patent ductus arteriosus (PDA)
5743	Valvular heart disease
18785	Heart septal defects
7894	Plastic repair of mitral valve
9498	Plastic repair of aortic valve
4548	Aortic valve disorders
22003	Regurgitation of unspecified heart valve
3418	Paroxysmal ventricular tachycardia
73476	Repair of defect of the atrioventricular septum
1005	Aortic regurgitation alone, cause unspecified
5477	Correction of tetralogy of Fallot
9450	Mitral valve regurgitation
2343	Aortic stenosis alone, cause unspecified
247	Congenital heart anomaly NOS
32163	Closure of defect of atrioventricular septum
2841	Implantation of intravenous cardiac pacemaker system
1294	Mitral valve prolapse
6401	H/O: heart disorder
18395	Other specified atrial septal defect
9286	Tricuspid regurgitation, cause unspecified
10187	Aortic regurgitation, non-rheumatic

	1735	Aortic aneurysm
	3520	Catheterisation of heart NOS
	7474	Ostium secundum atrial septal defect
	3625	Patent foramen ovale
	2427	Catheterisation of heart
	44896	Common atrioventricular-type ventricular septal defect
	9011	Eisenmenger's complex
	1536	Supraventricular tachycardia NOS
	2727	Patent ductus arteriosus
	561	Mitral regurgitation
	3255	Atrial septal defect NOS
	246	Ventricular septal defect
diabetes		
	14803	Diabetes mellitus, adult onset, no mention of complication
	85660	Diabetes type 1 review
	8403	Non-insulin dependent diabetes mellitus - poor control
	30323	Type 1 diabetes mellitus with persistent proteinuria
	30294	Type 1 diabetes mellitus with persistent microalbuminuria
	32359	Perceived control of insulin-dependent diabetes
	11626	Diabetic retinopathy NOS
	26054	Type 2 diabetes mellitus with persistent proteinuria
	2342	Diabetic neuropathy
	5884	NIDDM - Non-insulin dependent diabetes mellitus
	1682	Diabetes mellitus with ketoacidosis
	55239	Type 1 diabetes mellitus with gastroparesis
	18219	Type II diabetes mellitus
	18390	Type 2 diabetes mellitus with persistent microalbuminuria
	18824	Diabetic foot examination declined
	16230	Diabetes mellitus with neurological manifestation
	10692	Type 1 diabetes mellitus with ketoacidosis
	18278	Insulin treated Type 2 diabetes mellitus
	1407	Insulin treated Type 2 diabetes mellitus
	35383	Diabetic patient unsuitable for digital retinal photography
	17858	Type 1 diabetes mellitus
	11047	Conversion to insulin

	83532	Diabetes type 2 review
	18505	IDDM-Insulin dependent diabetes mellitus
	28769	Diabetic on insulin and oral treatment
	22823	Diabetic foot examination
	17859	Type 2 diabetes mellitus
	13074	Diabetic diet
	11471	Diabetes medication review
	1038	Insulin dependent diabetes mellitus
	22130	Diabetes monitoring default
	1647	Insulin dependent diabetes mellitus
	95994	Diabetic foot screen
	9974	Seen in diabetic eye clinic
	1323	Diabetic retinopathy
	506	Non-insulin dependent diabetes mellitus
	12675	Diabetes: shared care programme
	7563	Diabetic on diet only
	1549	Type 1 diabetes mellitus
	8836	Diabetes management plan given
	4513	Non-insulin dependent diabetes mellitus
	7069	Background diabetic retinopathy
	13067	Diabetic monitoring NOS
	1684	Diabetic on oral treatment
	2378	Diabetic - poor control
	12506	Diabetes: practice programme
	608	Follow-up diabetic assessment
	758	Type 2 diabetes mellitus
	711	Diabetes mellitus
	2379	Seen in diabetic clinic
	6125	Diabetic annual review
	3550	Diabetic monitoring
	9897	Diabetes monitoring admin.
epilepsy		
	9747	Epilepsy NOS
	4093	Status epilepticus
	5117	Grand mal status

	46603	Emergency epilepsy treatment since last appointment
	4801	Epileptic seizures - myoclonic
	22341	Epilepsy confirmed
	18471	Epileptic seizures - clonic
	1715	Epileptic absences
	8187	Tonic-clonic epilepsy
	6271	Status epilepticus, unspecified
	19550	Epilepsy control good
	8487	Myoclonic seizure
	5152	Epileptic seizures - tonic
	36696	Epilepsy monitoring NOS
	2907	Petit mal (minor) epilepsy
	3607	Fit (in known epileptic) NOS
	11752	Patient on maximal tolerated anticonvulsant therapy
	6983	Epilepsy monitoring
	9326	Epilepsy medication review
	11015	Seizure free >12 months
	573	Epilepsy
thyroid		
	11146	TSH - thyroid-stimulating hormone deficiency
	95885	Hypothyroidism annual review
	718	Subclinical iodine-deficiency hypothyroidism
	51481	Congenital hypothyroidism NOS
	24681	Thyroid dis.treatment changed
	10097	Congenital hypothyroidism
	28735	Hypothyroidism monitoring administration
	46630	Hypothyroidism monitoring second letter
	3941	Hypothyroidism NOS
	46057	Hypothyroidism monitoring first letter
	14704	Thyroid deficiency
	3290	Acquired hypothyroidism
	8268	Thyroid disease monitoring
	273	Hypothyroidism

Table A 3. List of ICD-10 codes

LRTI

B953	PROBABLE	Streptococcus pneumoniae as the cause of diseases classified to other chapters
J154	PROBABLE	Pneumonia due to other streptococci
J121	PROBABLE	Respiratory syncytial virus pneumonia
J152	PROBABLE	Pneumonia due to staphylococcus
J851	PROBABLE	Abscess of lung with pneumonia
J14X	PROBABLE	Pneumonia due to Haemophilus influenzae
J153	PROBABLE	Pneumonia due to streptococcus, group B
J852	PROBABLE	Abscess of lung without pneumonia
J158	PROBABLE	Other bacterial pneumonia
J168	PROBABLE	Pneumonia due to other specified infectious organisms
J218	PROBABLE	Acute bronchiolitis due to other specified organisms
J159	PROBABLE	Bacterial pneumonia, unspecified
J188	PROBABLE	Other pneumonia, organism unspecified
J151	PROBABLE	Pneumonia due to Pseudomonas
J129	PROBABLE	Viral pneumonia, unspecified
J209	PROBABLE	Acute bronchitis, unspecified
B961	PROBABLE	Klebsiella pneumoniae [K. pneumoniae] as the cause of diseases classified to other chapters
J157	PROBABLE	Pneumonia due to Mycoplasma pneumoniae
J13X	PROBABLE	Pneumonia due to Streptococcus pneumoniae
J210	PROBABLE	Acute bronchiolitis due to respiratory syncytial virus
J180	PROBABLE	Bronchopneumonia, unspecified
J219	PROBABLE	Acute bronchiolitis, unspecified
J189	PROBABLE	Pneumonia, unspecified
J181	PROBABLE	Lobar pneumonia, unspecified
J22X	PROBABLE	Unspecified acute lower respiratory infection

RTI

B349	PROBABLE	Viral infection, unspecified
R05X	POSSIBLE	Cough
R060	POSSIBLE	Dyspnoea

URTI

J398	PROBABLE	Other specified diseases of upper respiratory tract
J038	PROBABLE	Acute tonsillitis due to other specified organisms

J019	PROBABLE	Acute sinusitis, unspecified
J041	PROBABLE	Acute tracheitis
J040	PROBABLE	Acute laryngitis
J020	PROBABLE	Streptococcal pharyngitis
H660	PROBABLE	Acute suppurative otitis media
J208	PROBABLE	Acute bronchitis due to other specified organisms
J030	PROBABLE	Streptococcal tonsillitis
H709	PROBABLE	Mastoiditis, unspecified
J028	PROBABLE	Acute pharyngitis due to other specified organisms
J101	PROBABLE	Influenza with other respiratory manifestations, other influenza virus identified
H650	PROBABLE	Acute serous otitis media
H651	PROBABLE	Other acute nonsuppurative otitis media
H920	POSSIBLE	Otalgia
J00X	PROBABLE	Acute nasopharyngitis [common cold]
J029	PROBABLE	Acute pharyngitis, unspecified
H921	POSSIBLE	Otorrhoea
H659	PROBABLE	Nonsuppurative otitis media, unspecified
H669	PROBABLE	Otitis media, unspecified
J050	PROBABLE	Acute obstructive laryngitis [croup]
J039	PROBABLE	Acute tonsillitis, unspecified
J069	PROBABLE	Acute upper respiratory infection, unspecified

asthma

J458	Mixed asthma
J46X	Status asthmaticus
J450	Predominantly allergic asthma
J459	Asthma, unspecified

cardiac

Q232	Congenital mitral stenosis
Q264	Anomalous pulmonary venous connection, unspecified
Q238	Other congenital malformations of aortic and mitral valves
Q228	Other congenital malformations of tricuspid valve
Q201	Double outlet right ventricle
Q262	Total anomalous pulmonary venous connection

Q258	Other congenital malformations of great arteries
Q268	Other congenital malformations of great veins
I052	Mitral stenosis with insufficiency
Q282	Arteriovenous malformation of cerebral vessels
I510	Cardiac septal defect, acquired
Q254	Other congenital malformations of aorta
I059	Mitral valve disease, unspecified
I080	Disorders of both mitral and aortic valves
I083	Combined disorders of mitral, aortic and tricuspid valves
I081	Disorders of both mitral and tricuspid valves
Q208	Other congenital malformations of cardiac chambers and connections
I330	Acute and subacute infective endocarditis
Q251	Coarctation of aorta
I071	Tricuspid insufficiency
Q230	Congenital stenosis of aortic valve
I361	Nonrheumatic tricuspid (valve) insufficiency
I441	Atrioventricular block, second degree
Q244	Congenital subaortic stenosis
Q231	Congenital insufficiency of aortic valve
Q221	Congenital pulmonary valve stenosis
I341	Mitral (valve) prolapse
I440	Atrioventricular block, first degree
Q256	Stenosis of pulmonary artery
Q213	Tetralogy of Fallot
T825	Mechanical complication of other cardiac and vascular devices and implants
Z954	Presence of other heart-valve replacement
Q233	Congenital mitral insufficiency
I38X	Endocarditis, valve unspecified
I350	Aortic (valve) stenosis
Q219	Congenital malformation of cardiac septum, unspecified
T828	Other specified complications of cardiac and vascular prosthetic devices, implants and grafts
Q249	Congenital malformation of heart, unspecified
Q257	Other congenital malformations of pulmonary artery
T827	Infection and inflammatory reaction due to other cardiac and

		vascular devices, implants and grafts
	I351	Aortic (valve) insufficiency
	I517	Cardiomegaly
	I340	Mitral (valve) insufficiency
	Z952	Presence of prosthetic heart valve
	Q250	Patent ductus arteriosus
	I420	Dilated cardiomyopathy
	Q218	Other congenital malformations of cardiac septa
	Q211	Atrial septal defect
	Q210	Ventricular septal defect
	I270	Primary pulmonary hypertension
	Q212	Atrioventricular septal defect
	Z867	Personal history of diseases of the circulatory system
diabetes		
	E100	Insulin-dependent diabetes mellitus
	E105	Insulin-dependent diabetes mellitus
	E115	Non-insulin-dependent diabetes mellitus
	E106	Insulin-dependent diabetes mellitus
	E143	Unspecified diabetes mellitus
	Y423	Insulin and oral hypoglycaemic [antidiabetic] drugs
	E116	Non-insulin-dependent diabetes mellitus
	E112	Non-insulin-dependent diabetes mellitus
	E114	Non-insulin-dependent diabetes mellitus
	E111	Non-insulin-dependent diabetes mellitus
	E113	Non-insulin-dependent diabetes mellitus
	E108	Insulin-dependent diabetes mellitus
	O240	Diabetes mellitus in pregnancy: Pre-existing diabetes mellitus, insulin-dependent
	E139	Other specified diabetes mellitus
	E101	Insulin-dependent diabetes mellitus
	E109	Insulin-dependent diabetes mellitus
	E119	Non-insulin-dependent diabetes mellitus
epilepsy		
	Y466	Other and unspecified antiepileptics
	G408	Other epilepsy

	G419	Status epilepticus, unspecified
	G406	Grand mal seizures, unspecified (with or without petit mal)
	G403	Generalized idiopathic epilepsy and epileptic syndromes
	G409	Epilepsy, unspecified
thyroid		
	E02X	Subclinical iodine-deficiency hypothyroidism
	E038	Other specified hypothyroidism
	E890	Postprocedural hypothyroidism
	E031	Congenital hypothyroidism without goitre
	E039	Hypothyroidism, unspecified

Table A 4. Selection process of ethnicity codes from the CALIBER dataset

medid	readoricd10code	term	ethnicity
24690	9S7..00	Pakistani	Asian_or_Asian_British
12460	9i8..00	Pakistani or British Pakistani - ethnic category 2001 census	Asian_or_Asian_British
24740	9S8..00	Bangladeshi	Asian_or_Asian_British
28888	9i9..00	Bangladeshi or British Bangladeshi - ethn categ 2001 census	Asian_or_Asian_British
47997	9S42.12	Black West Indian	Asian_or_Asian_British
12482	9S6..00	Indian	Asian_or_Asian_British
12414	9i7..00	Indian or British Indian - ethnic category 2001 census	Asian_or_Asian_British
25937	9iFD.00	Iranian - ethnic category 2001 census	Asian_or_Asian_British
12653	9iA8.00	British Asian - ethnic category 2001 census	Asian_or_Asian_British
12420	9iF2.00	Filipino - ethnic category 2001 census	Asian_or_Asian_British
56127	9iF5.00	Hindu - ethnic category 2001 census	Asian_or_Asian_British
12473	9iF1.00	Japanese - ethnic category 2001 census	Asian_or_Asian_British
64133	9iA2.00	Kashmiri - ethnic category 2001 census	Asian_or_Asian_British
12730	9iF3.00	Malaysian - ethnic category 2001 census	Asian_or_Asian_British
26379	9SA8.00	Other Asian (NMO)	Asian_or_Asian_British
12513	9iA..00	Other Asian background - ethnic category 2001 census	Asian_or_Asian_British
12668	9SH..00	Other Asian ethnic group	Asian_or_Asian_British
28935	9iAA.00	Other Asian or Asian unspecified ethnic category 2001 census	Asian_or_Asian_British
12608	9iA4.00	Sri Lankan - ethnic category 2001 census	Asian_or_Asian_British
25411	9SC..00	Vietnamese	Asian_or_Asian_British
47950	9S42.11	Black Caribbean	Black_or_Black_British
12432	9iB..00	Caribbean - ethnic category 2001 census	Black_or_Black_British
12350	9iC..00	African - ethnic category 2001 census	Black_or_Black_British
35412	9S44.00	Black - other African country	Black_or_Black_British
12778	9S3..00	Black African	Black_or_Black_British
46812	9S43.11	Black North African	Black_or_Black_British
25451	9iFF.00	Moroccan - ethnic category 2001 census	Black_or_Black_British
32886	9iD1.00	Nigerian - ethnic category 2001 census	Black_or_Black_British
47028	9iFA.00	North African - ethnic category 2001 census	Black_or_Black_British
12443	9iD0.00	Somali - ethnic category 2001 census	Black_or_Black_British
12452	9S41.00	Black British	Black_or_Black_British
40097	9iD2.00	Black British - ethnic category 2001 census	Black_or_Black_British
57753	9S45.11	Black East African Asian	Black_or_Black_British
24339	9S4..00	Black, other, non-mixed origin	Black_or_Black_British
47969	9SA5.00	Other African countries (NMO)	Black_or_Black_British
32165	9S52.00	Other Black - Black/Asian orig	Black_or_Black_British
32389	9iD..00	Other Black background - ethnic category 2001 census	Black_or_Black_British
46047	9iD4.00	Other Black or Black unspecified ethnic category 2001 census	Black_or_Black_British
32136	9SG..00	Other black ethnic group	Black_or_Black_British
24962	9SA4.00	N African Arab/Iranian (NMO)	Black_or_Black_British
35350	9S47.00	Black - other Asian	Black_or_Black_British
24272	9S9..00	Chinese	Chinese_or_Other_Group
12468	9iE..00	Chinese - ethnic category 2001 census	Chinese_or_Other_Group
42290	9i2E.00	Gypsy/Romany - ethnic category 2001 census	Chinese_or_Other_Group
12434	9iF..00	Other - ethnic category 2001 census	Chinese_or_Other_Group
41214	9SAD.00	Other ethnic NEC (NMO)	Chinese_or_Other_Group
12757	9SJ..00	Other ethnic group	Chinese_or_Other_Group
30280	9SA..00	Other ethnic non-mixed (NMO)	Chinese_or_Other_Group
32126	9SAB.11	Turkish (NMO)	Chinese_or_Other_Group
12746	9i29.00	Turkish - ethnic category 2001 census	Chinese_or_Other_Group
32066	9SAB.00	Turkish/Turkish Cypriot (NMO)	Chinese_or_Other_Group
25676	9S5..00	Black - other, mixed	Mixed
32443	9SB6.00	Black African and White	Mixed
32425	9SB5.00	Black Caribbean and White	Mixed
12351	9i0..00	British or mixed British - ethnic category 2001 census	Mixed
12706	9i63.00	Chinese and White - ethnic category 2001 census	Mixed
47077	9iA3.00	East African Asian - ethnic category 2001 census	Mixed
46056	9iA9.00	Mixed Asian - ethnic category 2001 census	Mixed
25623	9S51.00	Other Black - Black/White orig	Mixed
12873	9i6..00	Other Mixed background - ethnic category 2001 census	Mixed
12591	9i2T.00	Other White or White unspecified ethnic category 2001 census	Mixed
32401	9SB2.00	Other ethnic, Asian/White orig	Mixed
12696	9SB..00	Other ethnic, mixed origin	Mixed
32420	9SB4.00	Other ethnic, other mixed orig	Mixed
12638	9i5..00	White and Asian - ethnic category 2001 census	Mixed
12437	9i4..00	White and Black African - ethnic category 2001 census	Mixed
12742	9i3..00	White and Black Caribbean - ethnic category 2001 census	Mixed
46059	9iF9.00	Arab - ethnic category 2001 census	Mixed
26246	9iFG.00	Latin American - ethnic category 2001 census	Mixed
12756	9iFH.00	South and Central American - ethnic category 2001 census	Mixed
26310	9S14.00	Other white British ethnic group	White
12681	9i22.00	Welsh - ethnic category 2001 census	White
12446	9S10.00	White British	White
98111	9i00.00	White British - ethnic category 2001 census	White
98111	9i00.00	White British - ethnic category 2001 census	White
26467	9S13.00	White Scottish	White
26391	9i2Q.00	Mixed Irish and other White - ethnic category 2001 census	White
24837	9S11.00	White Irish	White
12402	9i2R.00	Oth White European/European unsp/Mixed European 2001 census	White
12633	9SAC.00	Other European (NMO)	White
12421	9i2..00	Other White background - ethnic category 2001 census	White
12433	9i2G.00	Baltic Estonian/Latvian/Lithuanian - ethn categ 2001 census	White
28973	9i2H.00	Commonwealth (Russian) Indep States - ethn categ 2001 census	White
28866	9i2M.00	Croatian - ethnic category 2001 census	White
12355	9i27.00	Greek - ethnic category 2001 census	White
12769	9i28.00	Greek Cypriot - ethnic category 2001 census	White
46964	9iFC.00	Israeli - ethnic category 2001 census	White
12412	9i2B.00	Italian - ethnic category 2001 census	White
26341	9i2J.00	Kosovan - ethnic category 2001 census	White
12467	9i2F.00	Polish - ethnic category 2001 census	White
24270	9SA9.00	Irish (NMO)	White
12532	9i1..00	Irish - ethnic category 2001 census	White
12444	9S12.00	Other white ethnic group	White
22467	9S1..00	White	White
